# Novel nomograms predicting the survival of patients with nonsurgical thoracic esophageal squamous cell carcinoma treated with IMRT: A retrospective analysis

**DOI:** 10.1097/MD.0000000000030305

**Published:** 2022-10-07

**Authors:** Xingyu Du, Jing Dong, Ke Yan, Xiaobin Wang, Wenbin Shen, Shuchai Zhu

**Affiliations:** a Department of Radiation Oncology, The Fourth Hospital of Hebei Medical University, Shijiazhuang, Hebei, P.R. China.

**Keywords:** chemotherapy, esophageal cancer, nomogram, radiotherapy, systemic inflammation score

## Abstract

The purpose of this study was to evaluate several preradiotherapy serum inflammatory indicators, including the neutrophil to lymphocyte ratio (NLR), platelet to lymphocyte ratio (PLR), systemic immune-inflammation index (SII), and systemic inflammation score (SIS), and compare which of these indicators had the highest value in predicting survival. Inflammatory markers were combined with traditional prognostic factors, and novel nomogram models were developed to predict overall survival (OS) and progression-free survival (PFS) for patients with esophageal squamous cell carcinoma. A total of 245 patients were enrolled. The Kaplan–Meier method and univariate and multivariate analyses were used to compare survival differences. A total of 239 patients met the eligibility criteria. The survival numbers at 1, 3, and 5 years were 176, 83, and 62, respectively. The OS and PFS rates estimated at 1, 3, and 5 years were 74.6%, 36.8%, and 26.5% and 58.4%, 31.3%, and 20.5%, respectively. The differences in patients’ OS and PFS were significant when univariate analysis was applied based on inflammation-based measures. Multivariate analysis showed that tumor length, tumor stage, tumor/node/metastasis stage, chemotherapy, and SIS value were predictive variables for OS and PFS. The nomogram model established based on the multivariate models of the training data set had good predictive ability. The unadjusted C-index was 0.701 (95% CI, 0.662–0.740) and 0.695 (95% CI, 0.656–0.734) for OS and PFS, respectively. This study showed that the SIS-based nomogram could accurately predict the OS and PFS of patients with esophageal squamous cell carcinoma.

## 1. Introduction

Esophageal cancer is a relatively common malignant disease, with 455,800 newly diagnosed patients and 400,200 deaths due to this disease every year.^[1]^ Esophageal cancer is histologically classified into esophageal squamous cell carcinoma (ESCC), esophageal adenocarcinoma, and other subtypes. Historically, ESCC has been the main histological subtype, accounting for about 90% of esophageal cancers in East Asia, including China and Japan, and the incidence of ESCC has been increasing in some Asian countries. In China, the proportion of ESCC has exceeded 90%.^[2]^ Since this disease is usually diagnosed in the advanced stages and has rapid clinical progress, surgery is not an option for 40–60% of patients.^[3]^

It is difficult to accurately predict the prognosis of ESCC patients receiving radiotherapy (RT) due to the lack of extensive prospective studies. Some studies have reported a few survival-associated parameters, such as patients’ clinical symptoms and general state, tumor length, clinical stage, lymphatic/distant metastasis, and squamous cell carcinoma-related antigen levels, but they are not enough to evaluate patient outcomes.^[4–6]^ Therefore, it is important to develop a better predictive index for ESCC patient survival, which would be indispensable.

Immune inflammatory and nutritional indicators are closely related to the prognosis of many malignant tumors.^[7,8]^ Changes in neutrophil, lymphocyte, platelet, and monocyte levels have shown potential prognostic value. Various novel composite indicators have been calculated based on these peripheral blood parameters, including the neutrophil to lymphocyte ratio (NLR), platelet to lymphocyte ratio (PLR), lymphocyte to monocyte ratio (LMR), and some composite indexes, such as the systemic immune-inflammation index (SII), which involves neutrophils multiplied by platelets divided by lymphocytes.^[9]^ In addition, there are several methods of assessing nutritional status in cancer patients, with serum albumin measurements being one of the most commonly used methods.^[10]^ However, there is no scoring system that combines inflammatory and nutritional indicators and no accurate survival model to predict the prognosis of patients with ESCC.

The aims of this study were to compare the prognostic value of serum-based inflammatory and nutritional biomarkers for nonsurgical ESCC treated by RT or CRT and to establish a comprehensive and innovative nomogram model to assess the prognosis of ESCC patients.

## 2. Methods

### 2. 1. Patients

We retrospectively collected and analyzed pre-RT clinical and laboratory test data for 245 consecutive patients with thoracic ESCC who underwent radical RT or CRT at the Fourth Hospital of Hebei Medical University (Hebei, China) between January 2013 and December 2015. The inclusion criteria were: (i) pathologically confirmed ESCC before RT, (ii) Karnofsky Performance Scale score ≥ 70 points, (iii) patient refusal of surgery or no surgery for other reasons, (iv) no history of malignant disease and active double cancer at the time of esophageal cancer diagnosis, and (v) complete clinical and follow-up information available. The exclusion criteria were: (i) active hemorrhage or severe coagulation dysfunction, (ii) severe uncontrolled hypertension, (iii) severe cardiopulmonary diseases or abnormal liver and kidney function, (iv) death from other diseases during follow-up, (v) incomplete data, and (vi) patient lost to follow-up.

### 2. 2. Data collection

The limitation of the 2009 American Joint Committee on Cancer manual on the tumor/node/metastasis (TNM) staging criteria (7th edition) is that the criteria are only applicable to patients undergoing surgery alone, not to nonsurgical patients. Therefore, in this study, we classified tumor stage before RT according to the Clinical Classification of Esophageal Carcinoma Treated by nonsurgical Methods, the clinical prognostic value and practicability of which has been verified.^[11,12]^

All serum parameters were collected within 7 days before RT. These parameters included neutrophil, lymphocyte, monocyte, and platelet counts and albumin levels. NLR, PLR, LMR, SII, and systemic inflammation score (SIS) were calculated as follows: NLR = neutrophil count/lymphocyte count, PLR = platelet count/lymphocyte count, LMR = lymphocyte count/monocyte count, SII = neutrophil count × platelet count/lymphocyte count, decreased serum albumin and decreased LMR (<40 g/L and ≤ 4.15, respectively) = SIS 2, decreased serum albumin or decreased LMR = SIS 1, and elevated serum albumin and elevated LMR (≥40 g/L and > 4.15, respectively) = SIS 0.The optimal cutoff value was calculated based on the receiver operating characteristic (ROC) curve. All patients in this study signed informed consent forms, and all protocols in this study were approved by the ethics committee of the Fourth Hospital of Hebei Medical University.

### 2. 3. Follow-up

Using our computer database, clinical data were recorded and the date of death due to any cause was calculated for each patient. Patients who survived were included in our survival analyses. Medical history, physical examination, laboratory examination, electrocardiography, abdominal ultrasonography, esophageal barium meal, and chest computed tomography (CT) were performed every 3 months in the first 2 years and then every 6 months. Patients who were lost to follow-up were cut off at the last follow-up.

### 2. 4. Radiotherapy

A total of 239 patients with ESCC received intensity-modulated radiotherapy (IMRT) (95% PTV/ 50 to 66 Gy/ 25 to 33F, 1.8–2.0 Gy/ F, 5F/ W). The image data from the CT simulation scan were transmitted to the treatment planning system (Pinnacle 9.2, Philips Radiation Oncology System, USA), which was used to delineate the primary tumor area, enlarged lymph nodes, and at-risk organs. The gross tumor volume, clinical tumor volume (CTV), and planned tumor volume (PTV) were outlined according to the criteria issued by the National Comprehensive Cancer Network. The gross tumor volume included the esophagus with a thickened wall as shown by CT, esophageal film, and esophagoscopy, as well as swollen lymph nodes with a short axis diameter of ≥ 1 cm. The CTV was 0.5–0.8 cm extended from the gross tumor volume to the front, back, left, and right and 2.5–3.0 cm extended from the top and bottom, plus the corresponding lymphatic drainage area. The PTV was 0.5–0.6 cm extended from the CTV in all directions. The treatment plan included the prescribed dose of 50 to 66 Gy/ 25 to 33F, 1.8–2.0 Gy/ F, 5F/ week, with a median dose of 61.2 Gy. The total lungs V5 was ≤ 60%, V20 was ≤ 30%, V30 was ≤ 20%, and the lung mean dose was ≤ 16 Gy. The heart V25 was ≤ 50%, V40 was ≤ 30%, and the heart mean dose was ≤ 30 Gy. The maximum spinal cord dose was < 45 Gy. The irradiated field of esophageal lymph drainage was defined as CTV1. The scope of CTV1 varied with the location of the local esophageal lesion. Based on CTV1, PTV1 extended uniformly from 0.5–0.8 cm to the periphery. The required prescription dose of 95% PTV1 was 46 to 54 Gy, and 95% PTV and 95% PTV-nd were 50 to 66 Gy, with 1.80 to 2.06 Gy per fraction and 5 fractions weekly.

### 2. 5. Chemotherapy

Some patients had used several chemotherapy regimens. The most used regimen was cisplatin plus 5-fluorouracil (5-FU). Two cycles of cisplatin (75 mg/m²) on day 1 and 5-FU (800 mg/m²) on days 1 to 4 at were given at 4-week intervals. As for consolidation chemotherapy, 2 cycles of cisplatin (75 mg/m²) on day 1 and 5-FU (800 mg/m²) on days 1 to 4 were given starting approximately 4 weeks after CRT. The second chemotherapy regimen was docetaxel plus carboplatin. Docetaxel (7.5 mg/m²) on day 1 and carboplatin (area under the curve [AUC] = 2) on day 1 was used, and the patients received 4–6 cycles of chemotherapy. The third chemotherapy regimen was docetaxel plus cisplatin. The dosage and administration schedule was intravenous docetaxel (7.5 mg/m²) on day 1 and a continuous infusion of cisplatin (75 mg/m²) on day 1. The chemotherapy cycles were the same as those for docetaxel plus carboplatin.

## 3. Statistical analysis

GraphPad Prism version 8.0 and R version 4.0.3 (R Foundation for Statistical Computing) were used for statistical analysis. Continuous variables were compared using the unpaired t-test or Mann-Whitney nonparametric test. The chi-squared test and Fisher exact test were used to compare categorical variables. Survival analysis was conducted using the Kaplan–Meier method and the log-rank test. Multivariate analysis was performed using the Cox regression model for variables found to be significant among the univariate analysis, and the corresponding 95% confidence intervals (CIs) were calculated. A 2-tailed *P*-value < 0.05 was considered statistically significant.

ROC curves were calculated to determine the optimal cutoff value and assess the predictive power of the inflammation-based indicators for long-term survival. A nomogram for the OS and PFS probabilities at 1, 3, and 5 years was constructed based on multivariate Cox proportional hazards regression models. The distinguishing performance of the OS and PFS nomogram was evaluated by calculating the Harrell concordance index (C-index) value. In addition, we assessed the calibration of the nomogram to compare the estimated risk of the nomogram with the observed risk. The calibration chart illustrated the nomogram calibration of OS and PFS for 1, 3, and 5 years.

## 4. Results

### 4. 1. Demographics and clinical characteristics

From 245 ESCC patients, 4 cases were excluded due to incomplete peripheral blood indicator information, and 2 cases were excluded because they were lost to follow-up. A total of 239 patients met the eligibility criteria for analysis. The survival numbers at 1, 3, and 5 years were 176, 83, and 62, respectively. The baseline characteristics of all patients are summarized in Table 1. The median age was 67 years (range, 41 to 90 years). There were 140 (58.6%) male patients and 99 (41.4%) female patients. The median tumor length was 6.0 cm (range, 2.35–15.9 cm). The most common tumor location was the middle thoracic area, which affected 104 (43.5%) patients; this was followed by the upper thoracic area, which affected 80 (33.5%) patients, and then the lower thoracic area, which affected 55 (23.0%) patients. Regarding TNM stage, 210 patients (87.9%) were in stage II/III. The numbers of patients with tumor stages T1 + 2, T3, and T4 were 68 (28.5%), 83 (34.7%), and 88 (36.8%), respectively. In terms of N stage, 85 (35.6%) patients were N0, 109 (45.6%) were N1, and 45 (18.8%) were N2. All patients received treatment modalities, with 110 (46.0%) receiving IMRT alone and 129 (54%) receiving CRT. Involved-field irradiation was used for 139 (58.2%) patients while elective nodal irradiation was used for 100 (41.8%). There were significantly more patients with SIS = 1 (115, 48.2%) than with SIS = 0 (62, 25.9%) and SIS 1 (62, 25.9%).

**Table 1:** The characteristics of 239 patients with ESCC.

Characteristics	Cases (numbers, %)
Gender, male/ female	140 (58.6)/99 (41.4)
Age (years), ≤67/>67	127 (53.1)/112 (46.9)
Tumor location, upper/ middle/ lower	80 (33.5)/104 (43.5)/55 (23.0)
Tumor length (cm), ≤6.0/>6.0	121 (50.6)/118 (49.4)
T stage, 1 + 2/ 3/ 4	68 (28.5)/83 (34.7)/88 (36.8)
N stage, 0/ 1/ 2	85 (35.6)/109 (45.6)/45 (18.8)
TNM stage, I/ II/ III	29 (12.1)/97 (40.6)/113 (47.3)
CRT, no/ yes	110 (46.0)/129 (54.0)
Radiotherapy modalities, IFI/ ENI	139 (58.2)/100 (41.8)
prescription RT dose (Gy), ≤61.2/≥ 61.2	135 (56.5)/104 (43.5)
NLR, ≤2.24/ >2.24	80 (33.5)/159 (66.5)
PLR, ≤148/ >148	122 (51.0)/117 (49.0)
LMR, ≤4.15/ >4.15	155 (64.9)/84 (35.1)
Albumin, <40/ ≥40	152 (63.6)/87 (36.4)
SII, ≤738/ >738	144 (60.3)/95 (39.7)
SIS, 0/1/ 2	62 (25.9)/115 (48.2)/62 (25.9)

CRT = Chemoradiotherapy, ENI = Elective nodal Irradiation, IFI = Involved-Field Irradiation, LMR lymphocyte-to-monocyte ratio, NLR = neutrophil-to-lymphocyte ratio, PLR = platelet-to-lymphocyte, RT = radiotherapy, SII = systemic immune-inflammatory index, SIS = systemic inflammation score.

### 4. 2. OS and prognostic factors

**Figure 1.**
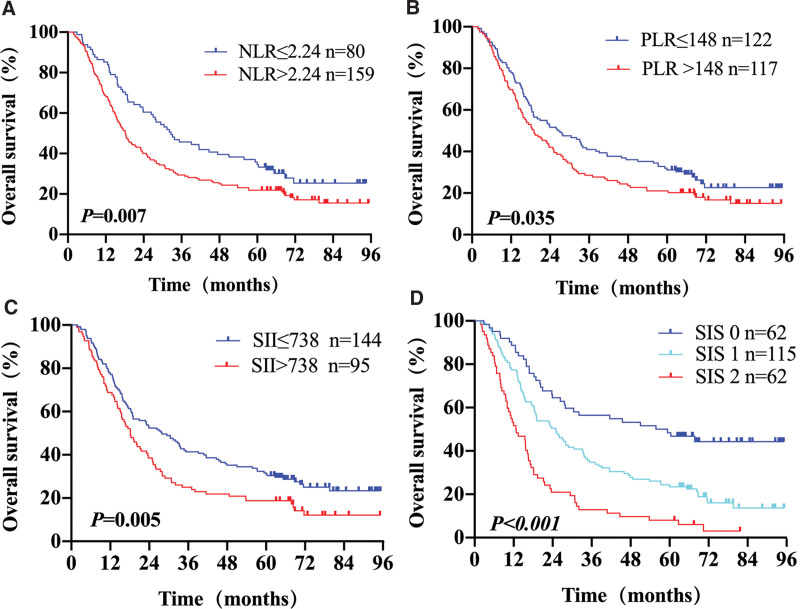
Kaplan–Meier survival curves of OS for different predicting parameters models: (A) NLR, (B) PLR, (C) SII, (D) SIS.

In all patients, the 1-, 3-, and 5-year OS rates were 74.6%, 36.8%, and 26.5%, respectively. The median OS was 21.6 months (range, 1.7–95.3 months). The correlations between the inflammation-based indexes and OS are shown in Figure 1. The results revealed that increases in NLR, PLR, SII, and SIS were associated with decreased OS. Univariate analysis results showed significant survival differences in terms of tumor location, tumor length, T stage, N stage, TNM stage, chemotherapy, RT modality, RT dose prescription, NLR (*P* = .007, Fig. 1A), PLR (*P* = .035, Fig. 1B), SII (*P* = .005, Fig. 1C), and SIS (*P* < .001, Fig. 1D) (Table 2). The different SIS grades were negatively correlated with OS. Multivariate regression analysis was performed to identify parameters independently related to OS. The results illustrated that SIS was an independent prognostic predictor of OS in ESCC patients (HR, 1.480; 95% CI, 1.153–1.900; *P* = .002). Other established predictors included tumor length (*P* < .001), T stage (*P* = .001), TNM stage (*P* = .003), and chemotherapy (*P* = .001) (Table 2).

**Table 2:** Univariate and multivariate analyzed of OS in patients with ESCC.

Characteristics	Univariate Analysis		Multivariate Analysis	
	HR (95% CI)	*P*-value	HR (95% CI)	*P*-value
Gender				
Male	1			
Female	0.975 (0.729–1.304)	0.864		
Age (years)				
≤67	1			
>67	1.002 (0.753–1.335)	0.988		
Tumor location				
Upper	1		1	
Middle	1.374 (0.980–1.926)	0.066	1.022 (0.692–1.510)	0.912
Lower	1.701 (1.152–2.511)	0.008	1.236 (0.791–1.933)	0.352
Tumor length (cm)				
≤6.0	1		1	
>6.0	2.404 (1.793–3.224)	<0.001	1.699 (1.168–2.470)	0.006
T stage				
1 + 2	1		1	
3	2.194 (1.496–3.217)	<0.001	1.284 (0.830–1.987)	0.262
4	2.122 (1.450–3.105)	<0.001	1.345 (0.993–1.897)	0.054
N stage				
0	1		1	
1	1.160 (0.832–1.619)	0.381	0.996 (0.689–1.441)	0.984
2	2.107 (1.414–3.138)	<0.001	1.098 (0.649–1.857)	0.729
TNM stage				
I	1		1	
II	2.051 (1.134–3.712)	0.018	1.884 (0.923–3.846)	0.082
III	3.911 (2.189–6.984)	<0.001	4.422 (1.948–10.038)	<0.001
CRT				
No	1		1	
Yes	0.610 (0.457–0.813)	0.001	0.669 (0.485–0.923)	0.014
Radiotherapy modalities				
IFI	1		1	
ENI	0.598 (0.443–0.806)	0.001	0.757 (0.524–1.092)	0.136
prescription RT dose (Gy)				
≤61.2	1		1	
>61.2	0.736 (0.549–0.986)	0.040	0.978 (0.702–1.363)	0.896
NLR				
≤2.24	1		1	
>2.24	1.526 (1.117–2.085)	0.007	0.948 (0.616–1.459)	0.808
PLR				
≤148	1		1	
>148	1.359 (1.020–1.811)	0.035	1.170 (0.814–1.681)	0.397
SII				
≤738	1		1	
>738	1.516 (1.135–2.026)	0.005	1.111 (0.743–1.662)）	0.609
SIS				
0	1		1	
1	1.970 (1.318–2.945)	0.001	1.937 (1.229–3.054)	0.004
2	3.370 (2.190–5.185)	<0.001	2.908 (1.678–5.041)	<0.001

CRT = Chemoradiotherapy, ENI =Elective nodal Irradiation, RT =radiotherapy, IFI = Involved-Field Irradiation, LMR lymphocyte-to-monocyte ratio, NLR = neutrophil-to-lymphocyte ratio, PLR = platelet-to-lymphocyte, SII = systemic immune-inflammatory index, SIS systemic inflammation score.

### 4. 3. PFS and prognostic factors

**Figure 2.**
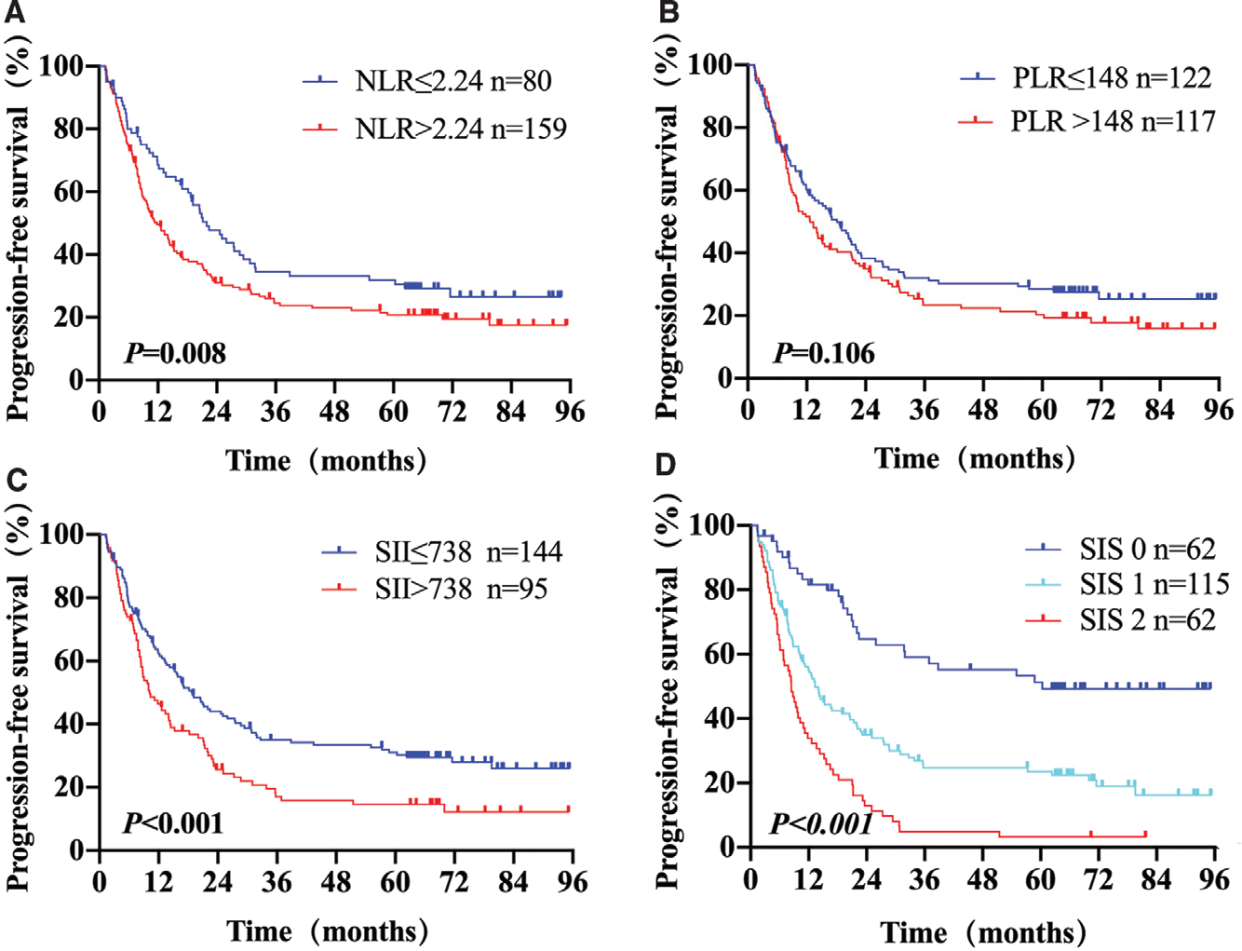
Kaplan–Meier survival curves of PFS for different predicting parameters models: (A) NLR, (B) PLR, (C) SII, (D) SIS.

The estimated 1-, 3-, and 5-year PFS rates for all patients were 58.4%, 31.3%, and 20.5%, respectively. The median PFS was 14.1 months (range, 1.3–95.3 months). The correlations between the inflammation-based indexes and PFS are shown in Figure 2. Increases in NLR (*P* = .008, Fig. 2A), SII (*P* < .001, Fig. 2C), and SIS (*P* < .001, Fig. 2D) were associated with reduced PFS. However, PLR failed to distinguish patients with longer PFS from those with shorter PFS (Fig. 2B). Univariate survival analysis results revealed significant associations between unfavorable PFS and higher NLR, SII, and SIS (Table 3). Other significant parameters related to PFS included tumor location, tumor length, T stage, N stage, TNM stage, chemotherapy, RT modality, and RT dose prescription (*P <* .05, Table 3). Multivariate Cox proportional hazards analysis showed that TNM stage (HR, 1.538; 95% CI, 1.045–2.266; *P* = .029), chemotherapy (HR, 0.639; 95% CI, 0.464–0.880; *P* = .006), and SIS (HR, 1.615; 95% CI, 1.249–2.088; *P* < .001) were independent predictors of PFS (Table 3).

**Table 3:** Univariate and multivariate analyses of PFS in patients with ESCC.

Characteristics	Univariate Analysis		Multivariate Analysis	
	HR (95% CI)	*P*-value	HR (95% CI)	*P*-value
Gender				
Male	1			
Female	1.010 (0.749–1.363)	0.947		
Age (years)				
≤67	1			
Tumor length (cm)				
≤6.0	1		1	
>6.0	1.198 (1.122-1.279)	<0.001	1.534 (1.029-2.285)	0.036
T stage				
1 + 2	1		1	
3	1.944 (1.312-2.880)	0.001	1.111 (0.709-1.740)	0.645
4	2.022 (1.377-2.969)	<0.001	1.557 (0.907-1.911)	0.054
N stage				
0	1		1	
1	1.293 (0.914-1.830)	0.147	1.205 (0.789-1.842)	0.388
2	2.243 (1.484-3.391)	<0.001	1.582 (0.872-2.871)	0.131
TNM stage				
I	1		1	
II	2.010 (1.107-3.651)	0.022	2.065 (0.998-4.275)	0.050
III	3.497 (1.952-6.265)	<0.001	3.665 (1.574-8.685)	0.003
CRT				
No	1		1	
Yes	0.620 (0.460-0.834)	0.002	0.651 (0.466-0.909)	0.012
Radiotherapy modalities				
IFI	1		1	
ENI	0.538 (0.394–0.735)	<0.001	0.745 (0.507-1.095)	0.135
prescription RT dose (Gy)				
≤61.2	1		1	
>61.2	0.655 (0.484-0.888)	0.006	0.908 (0.636-1.296)	0.594
NLR				
≤2.24	1		1	
>2.24	1.551 (1.121-2.147)	0.008	0.867 (0.551-1.365)	0.583
PLR				
≤148	1			
>148	1.276 (0.948-1.716)	0.106		
SII				
≤738	1		1	
>738	1.724 (1.278-2.324)	<0.001	1.337 (0.883-2.024)	0.170
SIS				
0	1		1	
1	2.501 (1.611-3.882)	<0.001	2.099 (1.278-3.449)	0.003
2	4.166 (2.622-6.620)	<0.001	2.418 (1.375-4.252)	0.002

CRT = Chemoradiotherapy, ENI = Elective nodal Irradiation, IFI = Involved-Field Irradiation, LMR lymphocyte-to-monocyte ratio, NLR = neutrophil-to-lymphocyte ratio, PLR = platelet-to-lymphocyte, RT = radiotherapy, SII = systemic immune-inflammatory index, SIS systemic inflammation score.

### 4. 4. Predictive value of inflammatory indexes

**Figure 3.**
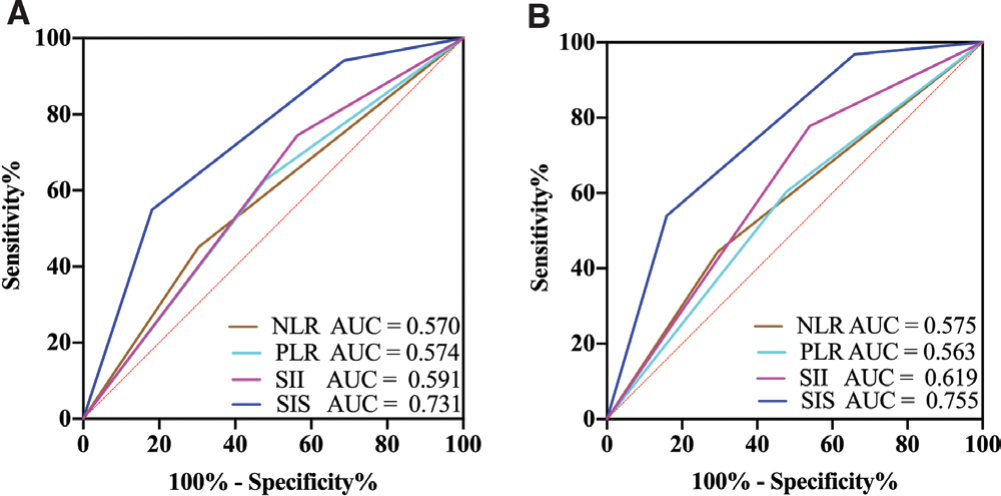
AUROC for OS (A) and PFS (B) stratifed by each inflammation-based index at 1-, 3-, and 5-year.

The areas under the ROC curves (AUROCs) were calculated through statistical analysis to estimate the predictive power of all inflammation-based indexes for both OS and PFS in ESCC patients, and the predictions were calculated with patients’ 5-year follow-up information. The results showed that the AUROC values of SIS were consistently higher than those of the other inflammatory indexes, with AUCs of 0.731 for OS and 0.755 for PFS (Fig. 3). Subsequently, a comparative study revealed that patients with higher SIS had unfavorable outcomes more frequently than those with lower SIS, and 1-, 3-, 5-year OS and PFS had statistically significant differences among the different SIS levels (Table 4).

**Table 4:** Comparison of the difference SIS effect on OS and PFS.

Time	OS (number, %)					PFS (number, %)				
	0 (n = 62)	1 (n = 115)	2 (n = 62)	X^2^	*P*-value	0 (n = 62)	1 (n = 115)	2 (n = 62)	X^2^	*P*-value
1y	55 (88.7)	89 (77.4)	32 (51.6)	23.58	<0.0001	48 (77.4)	61 (53.0)	21 (33.9)	23.86	<0.0001
3y	35 (56.5)	40 (34.8)	8 (12.9)	25.94	<0.0001	31 (50.0)	23 (20.0)	3 (4.8)	36.62	<0.0001
5y	30 (48.4)	27 (23.5)	5 (8.0)	26.94	<0.0001	26 (41.9)	21 (18.3)	2 (3.2)	29.18	<0.0001

OS = overall survival, PFS = progression-free survival, SIS = systemic inflammation score.

### 4. 5. Establishment and assessment of the nomogram model

**Figure 4.**
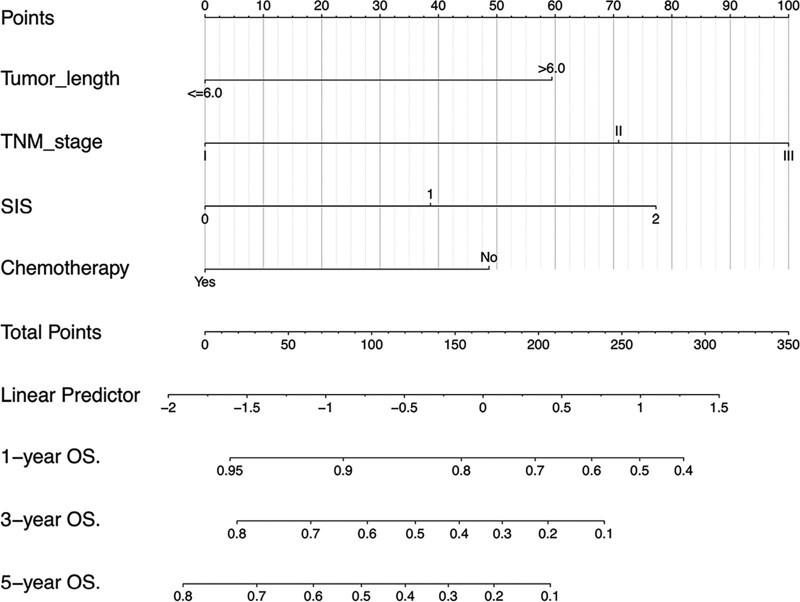
Nomogram for predicting 1-, 3-, and 5-year OS probabilities of patients with ESCC. The relevant scores of each factor in the nomogram are as follows: tumor length (0, 59 points), TNM stage (0, 100 points), SIS (0, 77 points), Chemotherapy (0, 48 points). The total score was obtained by adding the scores of each risk factor in the nomogram.

**Figure 5.**
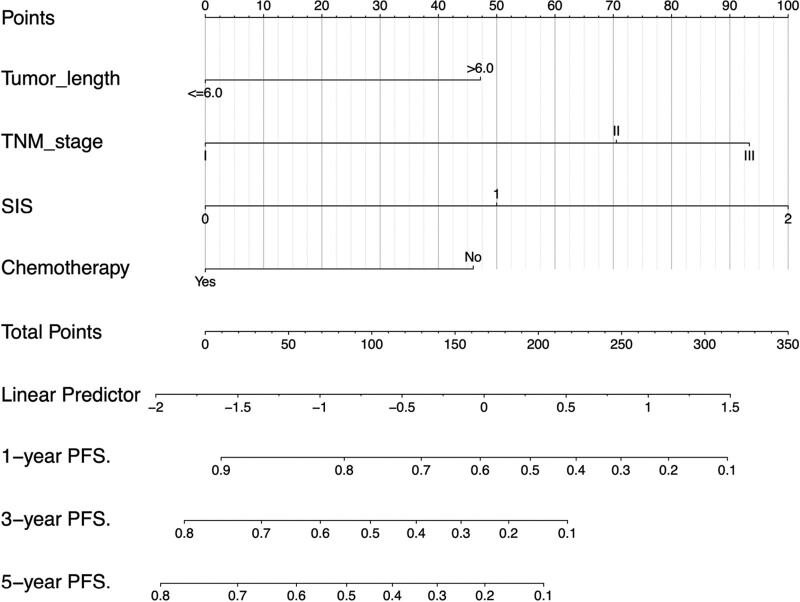
Nomogram for predicting 1-, 3-, and 5-year PFS probabilities of patients with ESCC. The relevant scores of each factor in the nomogram are as follows: tumor length (0, 47 points), TNM stage (0, 93 points), SIS (0, 100 points), chemotherapy (0, 46 points).The total score was obtained by adding the scores of each risk factor in the nomogram.

**Figure 6.**
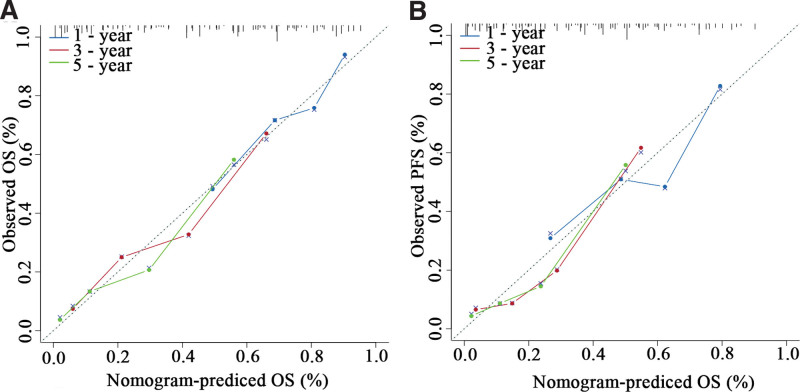
Calibration for predicting 1-, 3-, and 5-year OS (A), PFS (B).

**Figure 7.**
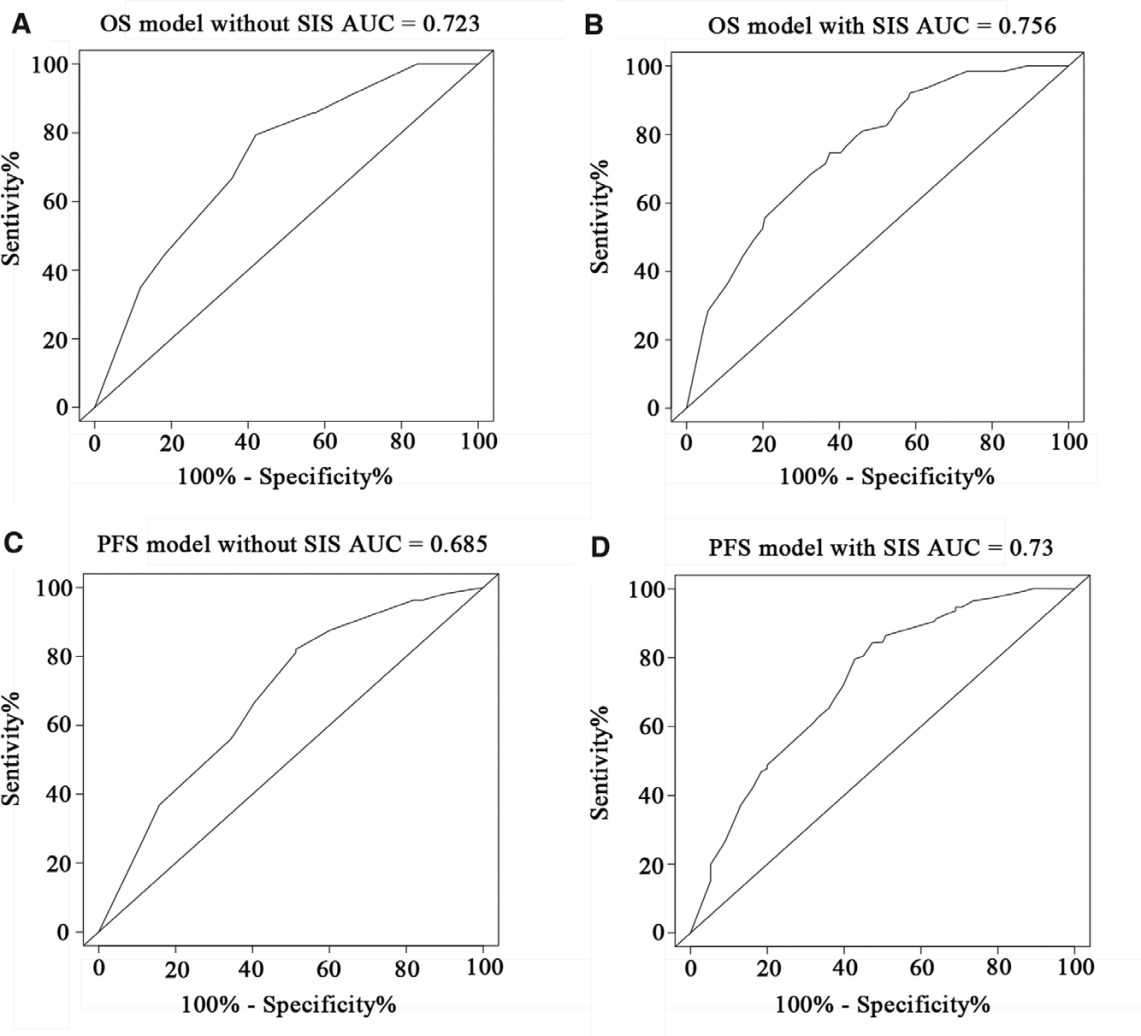
ROC curve analysis of the predictive value of the nomogram model with or without SIS. ROC = receiver operating characteristic; SIS = systemic inflammation score.

Based on the results of the multivariate Cox regression analysis, we used tumor length, TNM stage, chemotherapy, and SIS to construct the nomogram (Figs. 4 and 5). The total score of these prognostic factors could be used to determine the probabilities of OS and PFS at 1, 3, and 5 years. The performance of the nomogram was evaluated using the C-index and calibration curve. The C-index was 0.701 (95% CI, 0.662–0.740) for OS prediction and 0.695 (95% CI, 0.656–0.734) for PFS prediction. The calibration curve showed agreement between the standard curve and the nomogram prediction. The calibration curve also had good prediction consistency (Fig. 6). The ROC analysis results indicated that the model combining the SIS and other prognostic factors had more favorable predictive ability than the model without the SIS. The AUC of OS for the above models were 0.756 and 0.723, and the PFS were 0.73 and 0.685, respectively (Fig. 7 A-D).

## 5. Discussion

Several inflammatory factors are associated with long-term survival in ESCC patients.^[13–15]^ Although most relevant studies have demonstrated the prognostic effect of inflammatory markers in ESCC, so far, there has been no comparison of prognostic value among these markers, and no comprehensive prognostic model has been established. In this study, inflammatory markers were assessed and rigorous statistical methods were used. The nomogram model based on traditional prognostic indicators and SIS was considered to be a reliable method to generate more accurate prognostic predictions.

In this study, SIS 1 and SIS2 patients had higher chance to be of tumor length > 6.0 cm and TNM stage III, while SIS 0 patients usually had tumor length ≤ 6.0 cm and an early TNM stage. This result suggested that the immune inflammatory response of patients is correlated with disease progression. In addition, due to malnutrition, anorexia, or abnormal liver function, the albumin levels of patients with advanced disease were reduced. Patients with high SIS values tended to have a worse prognosis than those with low SIS values. In terms of treatment, SIS 0 patients preferred to combine chemotherapy, elective nodal irradiation, and a prescription dose > 61.2 Gy, while SIS 2 patients preferred IMRT alone, involved-field irradiation, and a prescription dose ≤ 61.2 Gy. This result suggested that patients with lower SIS could tolerate more active treatment and achieve better survival.

The efficacy of SIS in predicting other malignancies has been reported. Chang et al showed that SIS was an independent prognostic factor for OS in renal cell carcinoma and that SIS 2 was associated with a shorter median OS.^[16]^ Inokuchi et al reported that SIS was closely related to the prognosis of patients with hepatocellular carcinoma; the higher the SIS score, the worse the prognosis, showing that SIS was an important predictor of OS and PFS.^[17]^

The same results were obtained in our study. The lower the SIS, the higher the survival. Multivariate analysis results showed that SIS was closely correlated to clinical characteristics and was an independent factor for OS and PFS in ESCC patients. According to the AUROC, the OS and PFS AUCs of SIS (0.731 and 0.755, respectively) were higher than those of other inflammatory factors.

A low SIS indicated low LMR and/or albumin levels, which may increase nonspecific inflammation, immune system dysfunction, and malnutrition. However, when it comes to the clinical prognosis of ESCC, the mechanism of the predictive value of SIS is complicated and unclear.

The reason for the low level of LMR is the increase of monocytes and the decrease of lymphocytes. Monocytes promote tumorigenesis and angiogenesis through local immunosuppression and stimulation of tumor neovasculogenesis.^[18,19]^ This may explain why elevated monocyte counts can lead to poor clinical outcomes in patients with various types of cancers. Noriyuki et al reported that a low LMR value was a significant and independent predictor of cancer-specific survival in non-elderly patients with esophageal cancer.^[20]^

In addition, there is increasing evidence that lymphocytes are essential for the antitumor immune response owing to several mechanisms, including the abilities to enhance tumor cell apoptosis, inhibit tumor cell proliferation, and promote metastasis.^[21]^ Davuluri et al demonstrated that lymphocyte reduction during CRT for ESCC was associated with adverse outcomes and that lymphocytes play a role in host immunity when it comes to disease control.^[22]^ Zhang also confirmed that lymphatic invasion is an independent and favorable prognostic factor of disease-free survival in patients with non-small cell lung cancer who underwent lobectomy, lymph node dissection, and adjuvant chemotherapy.^[23]^

Additionally, serum albumin levels are generally used to assess nutritional status, disease severity, and disease progression in patients with various cancers, and serum albumin is considered an independent factor of survival.^[24]^ Malnutrition is closely related to the imbalance of the tumor microenvironment, and it can weaken a patient’s defense mechanisms, such as humoral immunity, cellular immunity, and phagocytosis, leading to the risk of infection and the poor efficacy of antitumor treatments.^[25]^ Since nutrition is an important determinant of immune response, reduced serum albumin levels reflect both malnutrition and a sustained systemic inflammation response.^[26]^

Thus, SIS, which is based on LMR and serum albumin levels, can be used to predict the prognosis of cancer. This observation provides a basis for prospective testing of chemotherapy and RT strategies that may have less impact on host immunity.

Finally, to better visualize the predictive power of SIS, we established nomogram models based on relevant predictive factors.^[27,28]^ Statistical analysis results showed that the models had accurate predictive capabilities. For example, one patient had a tumor length of 5 cm, TNM stage II, combined chemotherapy, and SIS 2 status; the OS nomogram model total points equaled 148 and the 1-, 3-, and 5-year OS rates were approximately 81%, 42%, and 29%, respectively, while the PFS nomogram model total points equaled 171 and the 1-, 3-, and 5-year PFS rates were 59%, 24%, and 18%, respectively. These nomogram models showed that SIS could provide additional prognostic information for traditional survival factors, which may help clinicians better optimize care and stratify patients for health care resources to thereby improve the prognosis of ESCC patients. Meanwhile, these models included conventional prognostic factors and SIS, which may help in the identification of a patient’s OS and PFS early on and allow them to receive timely intervention.

Nonetheless, the current research has some limitations. First, this study was a retrospective study with a single-center design. The number of patients assessed by these models was relatively small, and there was no external validation. Second, this study did not compare dynamic changes in peripheral blood inflammatory markers during and after IMRT or CRT. Therefore, prospective verification trials using a larger quantity of cases are needed to confirm our findings.

## 6. Conclusion

In conclusion, the predictive abilities of several inflammation-based prognostic factors were assessed and compared in patients with ESCC. SIS was independently associated with OS and PFS, and the nomogram model based on SIS had favorable predictive ability. SIS 0 patients have better OS and PFS, and SIS 1 and SIS 2 patients should receive stronger monitoring and more rigorous treatment to avoid tumor progression. However, further studies are necessary to validate the findings of this study and to explore the relevant mechanisms, which will ultimately provide greater evidence for individualized treatment of ESCC patients.

## Acknowledgments

We acknowledge the Medical Records Department of The Fourth Hospital of Hebei Medical University for collecting the survival data of the patients. We thank the patients who were included in this study. The authors have no conflicts of interest to disclose.
